# Myosin Va plays essential roles in maintaining normal mitosis, enhancing tumor cell motility and viability

**DOI:** 10.18632/oncotarget.17920

**Published:** 2017-05-17

**Authors:** Yan-Ruide Li, Ai Zhong, Han Dong, Lu-Han Ni, Fu-Qing Tan, Wan-Xi Yang

**Affiliations:** ^1^ The Sperm Laboratory, College of Life Sciences, Zhejiang University, Hangzhou, China; ^2^ The First Affiliated Hospital, College of Medicine, Zhejiang University, Hangzhou, China

**Keywords:** myosin Va, testicular cancer, prostate cancer, tumorigenesis, actin

## Abstract

Myosin Va, a member of Class V myosin, functions in organelle motility, spindle formation, nuclear morphogenesis and cell motility. The purpose of this study is to explore the expression and localization of myosin Va in testicular cancer and prostate cancer, and its specific roles in tumor progression including cell division, migration and proliferation. We detected myosin Va in testicular and prostate tumor tissues using sqRT-PCR, western blot, and immunofluorescence. Tumor samples showed an increased expression of myosin Va, abnormal actin and myosin Va distribution. Immunofluorescence images during the cell cycle showed that myosin Va tended to gather at cytoplasm during anaphase but co-localized with nucleus during other phases, suggesting the roles of myosin Va in disassembly of spindle microtubule, movement of chromosomes and normal cytokinesis. In addition, multi-nucleation and aberrant nuclear morphology were observed in myosin Va-knockdown cells. Wounding assay and CCK-8-based cell counting were conducted to explore myosin Va roles in cell migration, viability and proliferation. Our results suggest that myosin Va plays essential roles in maintaining normal mitosis, enhancing tumor cell motility and viability, and these properties are the hallmark of tumor progression and metastasis development. Therefore, an increased understanding of myosin Va expression and function will assist in the development of future oncodiagnosis and -therapy.

## INTRODUCTION

Male reproductive system diseases become a common concern, especially testicular cancer and prostate cancer. Testicular cancer is among the most common malignancies in young (20 to 34-year-old) men worldwide [1]. Numerous studies showed an increase of testicular cancer in the last 50 years with substantial differences among countries [1, 2]. Among the different kinds of testicular cancer, approximately 95% are germ cell tumors [3, 4]. Early cancer diagnosis, adequate surgical excision, and optimal adjuvant treatment are the most common ways of testicular cancer treatment at present. However, due to the heterogeneous characteristics of this cancer, it is difficult to diagnose and defeat this disease [5]. On the other hand, prostate cancer is estimated as the most common cancer in males and one of the major causes of cancer-related deaths in the United States in 2017 [6]. Screening by prostate-specific antigen values did not decrease prostate cancer mortality [7]. Radiation-involved treatment was likely to raise the risk of a radiation-induced second primary cancer [8]. As a result, Searching for the medicable factors and medicines to improve prognostic evaluation in patients with testicular and prostate cancer is of vital importance.

Tumorigenesis is a multifaceted process primarily involving changes in genetic or chromosomal stability, which subsequently disorganize a series of normal cell processes that cause the development of malignancy [9, 10]. During tumor progression, some alterations in cell morphology and physiology happen, including tumor cell proliferation, cell polarity loss, adhesion reduction, protrusion formation, cell invasion and metastasis development [11–14]. Accordingly, the molecules and factors involved in these processes are regarded as potential candidates identified as prognostic markers in testicular and prostate cancer. Interestingly, these processes involve the movements of cells or organelles [15]. Molecular depictions of cell migration have been illustrated in the past thirty years, which contain cytoskeletal alterations, motor protein (including myosin, kinesin and dynein) transportation and cell-matrix interactions [16]. Increasing progress has been made in the identification of molecular mechanisms underlying these cellular dynamic processes. Among them, the interplay between microfilament and myosins has received the attention of researchers.

Myosins are actin-based molecular motors which translocate along microfilaments, transport cargoes, and produce muscle force. These motor proteins convert energy from the hydrolysis of ATP to mechanical force [17]. At least fifteen distinct classes of myosins have been discovered in the past twenty years that play multiple cellular roles. Among the different classes, the functional units of myosins are similar [18]. Myosins are typically composed of three subdomains: the head domain is the center of power, which combines with actin filaments and ATP, producing mechanical stress [19]; the neck domain contains one or more light chain-binding motifs named IQ motifs, which have the consensus of IQXXXRGXXXR (where X is any kind of amino acid) and bind with calmodulin and the light chains of myosin [20, 21]; the divergent tail domains facilitate cargo binding and transportation. In addition, many kinds of myosins, including myosin V, have a coiled-coil α-helix structure in their tails, which allow dimer assembly and produce bipolar filaments [22].

**Figure 1:**
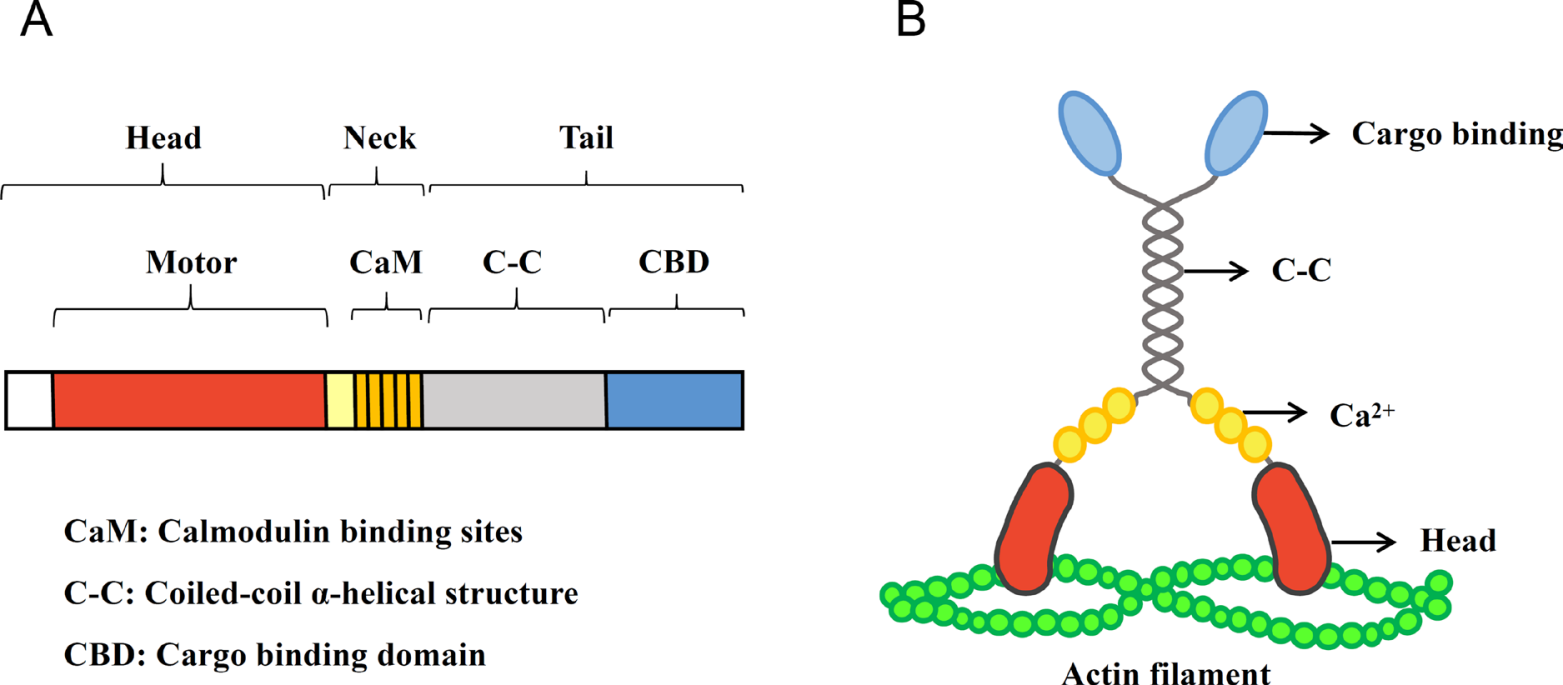
The domain structure and protein structure of myosin Va (**A**) The scale bar diagram represents the domain structure of myosin Va heavy chain sequence. Myosin Va is typically composed of three subdomains: the head, neck and tail domain. The head domain combines with actin filaments and ATP, producing mechanical stress (19), and the most amino terminal domain is the motor domain. The neck domain contains the putative essential light chain (ELC) and calmodulin binding sites (CaM) (20,21). The tail domain is divided into two regions: a region predicted to form a coiled-coil α-helix structure (C-C) and a carboxyl terminal globular domain, which is also called cargo-binding domain (CBD), taking part in the cargo binding and transportation (22). (**B**) The diagram shows the myosin Va structure. The distinct protein structures are expressed by the different genetic domains which are displayed in (A). Myosin Va is an actin-based molecular motor which translocates along microfilaments, transports cargo and produces muscle force. It converts energy from ATP hydrolysis to mechanical force.

Myosin V, as one of the best characterized and functionally diverse myosin groups, is implicated in mRNA transport, cell polarity, spindle-pole alignment and membrane trafficking [23]. Three most closely related gene sequences, including chicken brain myosin V, human myosin V and mouse dilute gene, are classified as myosin Va [24]. The genetic domain structure and protein structure of myosin Va have been demonstrated in Figure 1. Previous studies showed that during mitosis/meiosis, myosin Va participates in organelle motility [25], spindle formation [26] and nuclear morphogenesis [27]. Furthermore, an increasing pool of data indicates that myosin Va is also involved in other cellular cancerogenic functions such as proliferation and migration of cancer cells. The upregulation of myosin Va by Snail (a zinc finger transcriptional repressor binding to the promoters of cancer metastasis-related genes [28]) promotes cell invasion and metastasis of human colorectal cancer. After knocking-down the expression of myosin Va by RNAi, a decrease of cell migration velocity is clearly observed in lung cancer cells [29]. Myosin Va also mediates Bcl-xL [30], an anti-apoptotic protein which functions at ER-mitochondrion membrane to regulate bioenergetics [31]. Bcl-xL also affects tumor cell migration and invasion, and can be mediated by myosin Va to promote islet tumor cell motility [30].

In our previous paper, we put forward a completely new concept about ‘chromomyosin’ [11]. Myosins translocate along cytoplasmic microfilaments and transport cargoes, but lots of work show that myosins exist in nucleus and take part in nuclear related activities [11]. However, microfilaments don't diffusely distribute in the nucleus but display monomer existence [32]. As a result, these unconventional nuclear myosins, which exert functions effectively but don't interact with microfilaments, are defined as ‘chromomyosin’. Chromomyosin exists in nuclear matrix and interplays with nuclear monomer actin, playing important roles in cytokinesis, spindle assembly, nuclear material transportation [11] and RNA transcription [33]. In our present study, we proposed a hypothesis that myosin Va is a kind of chromomyosin and participates in nuclear related activities, including normal assembly of spindle microtubule, movement of chromosomes and normal cytokinesis.

The purpose of the present study is to investigate the functions of myosin Va during tumorigenesis. We intend to examine the role of myosin Va in mitosis, tumor cell migration, and proliferation. To address these questions, RT-PCR, western blot and immunofluorescence were used to examine myosin Va expression in testicular and prostate tissues, and siRNA knock-down was utilized for the analysis at cell-level. We used wound-healing migration assay to explore the association between myosin Va and tumor cell migration, and used CCK-8-based cell counting to find out its role in cell viability and proliferation. This study will help us to understand more about the functions of myosin Va, especially during tumorigenesis, and the main data may help doctors to perform accurate oncodiagnosis and anti-tumor therapies.

## RESULTS

### Histological observation of testicular and prostate tumor

**Figure 2:**
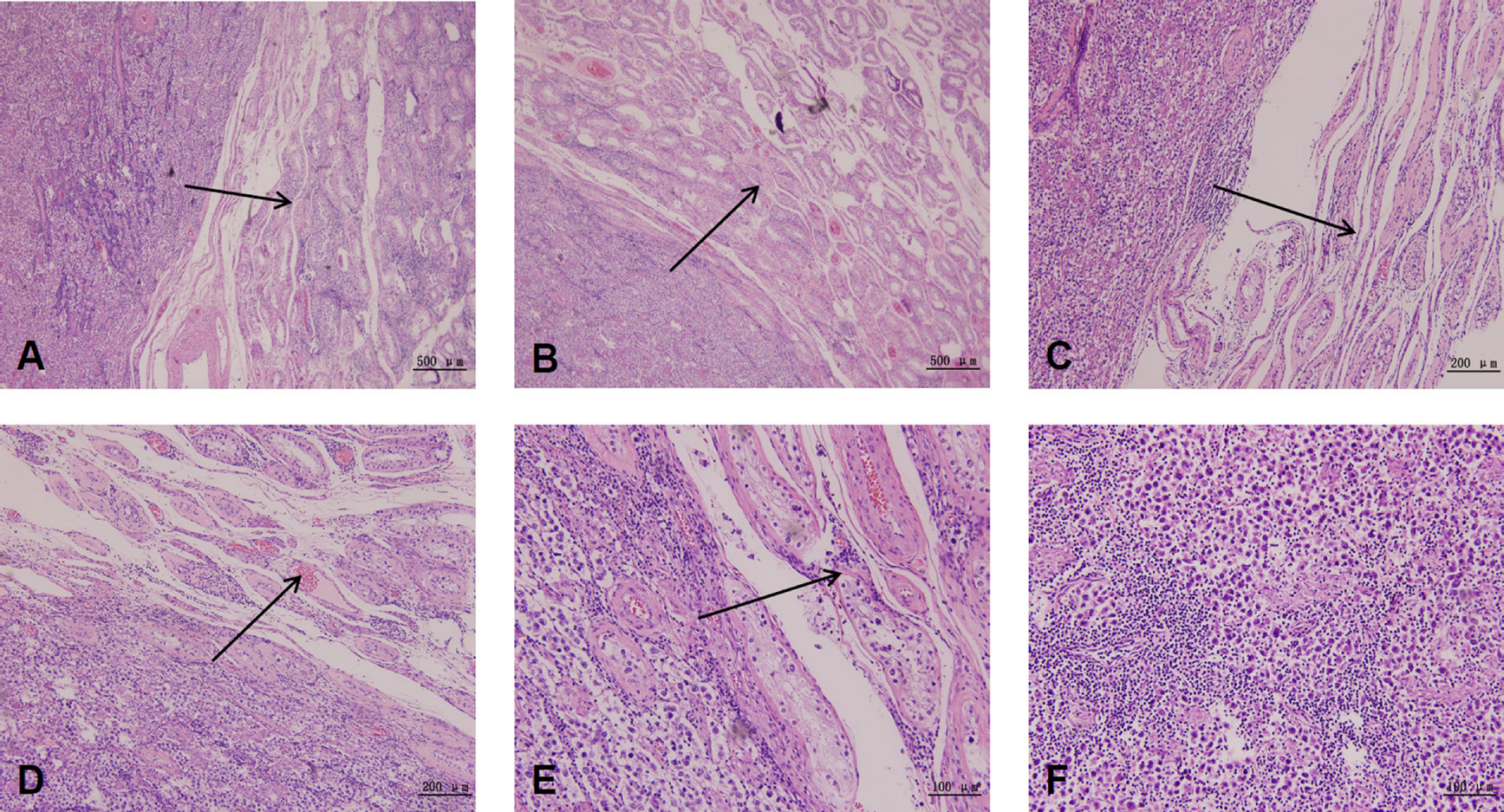
Histological examination of testicular cancer tissue Histological examination displays distinctive structures in testicular cancer tissue. Tumor cells gradually invade and replace the seminiferous tubules (**A**, **C**, **E**) and blood vessels (**B**, **D**) with fibrinoid material. The black arrows represent the direction and route of tumor cell invasion. These spindle tumor cells show a monophasic growth pattern, with abundant dense eosinophilic cytoplasm and pleomorphic hyperchromatic nuclei (**F**).

Histological examination indicated distinctive structures in testicular cancer tissues. Tumor cells gradually invaded and replaced the seminiferous tubules (Figure 2A, 2C and 2E) and blood vessels (Figure 2B and 2D) with fibrinoid material. These spindle tumor cells showed monophasic growth patterns, with abundant dense eosinophilic cytoplasm and pleomorphic hyperchromatic nuclei. Frequent mitoses took place in these epithelioid cells (Figure 2F). High-density testicular tumor cells filled up the whole view, and just a few extracellular matrices remained.

**Figure 3:**
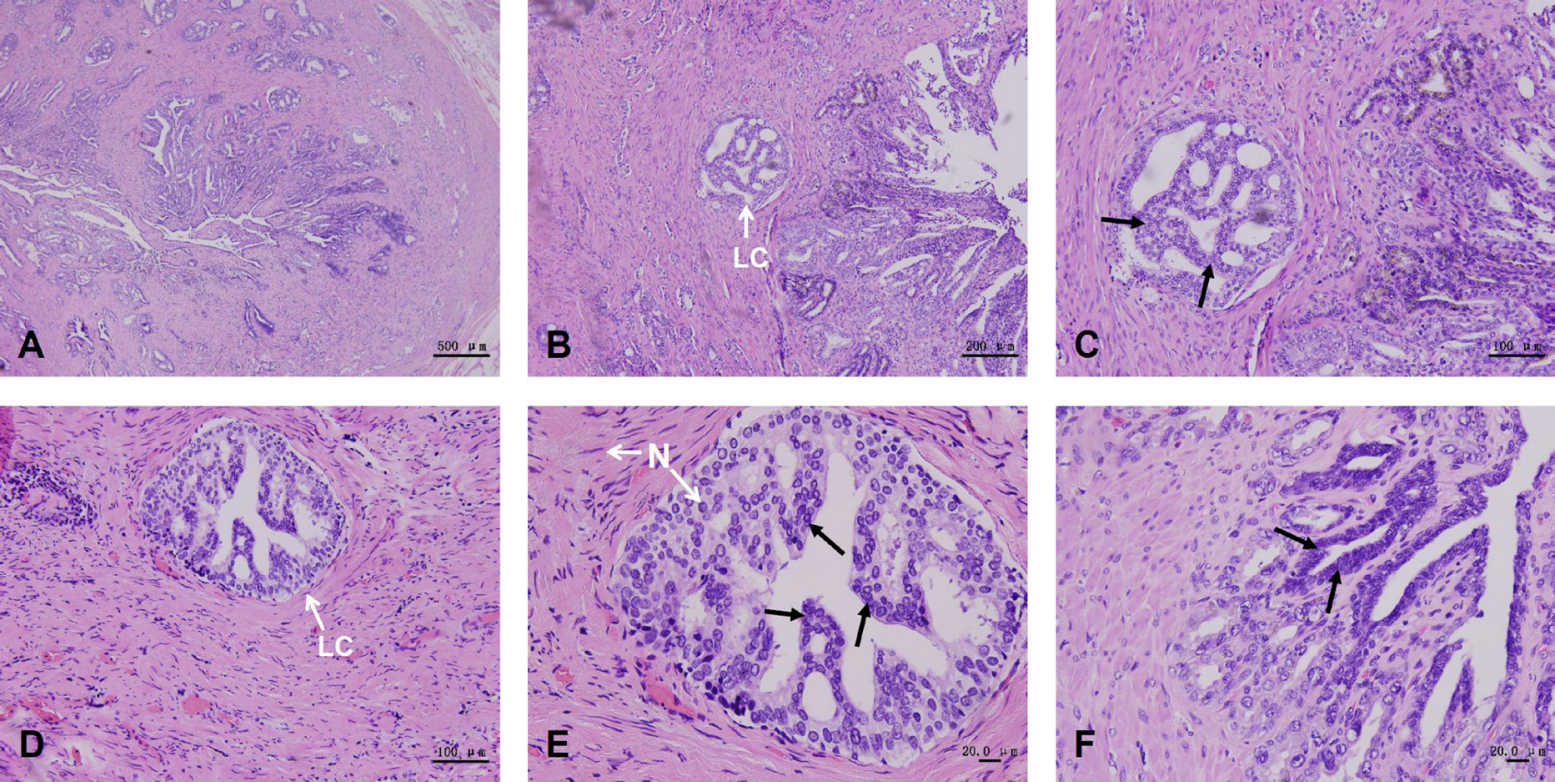
Histological examination of prostate cancer tissue These results show the characteristics of prostatic intraepithelial neoplasia (PIN). Prostate cancer tissues form irregular glands, presenting small cancer cells and deep nuclear staining (**A**). Luminal cells with persistence of the basal layer and with no basal membrane rupture present neoplastic nuclear atypia (**B**). Several patterns of prostate cancer, such as cribriform (**C**), tufting (**D**, **E**) and flat (**F**) are observed in this assay. Black arrows indicate neoplastic nuclear atypia of the luminal cells. N, nucleus. LC, luminal cells.

The histological examination results of prostate cancer showed characteristics of prostatic intraepithelial neoplasia (PIN). Luminal cells presented neoplastic nuclear atypia, with persistence of basal layer and with no evidence of basal membrane rupture (Figure 3A and 3B). Several patterns of prostate cancer, such as cribriform (Figure 3C), tufting (Figure 3D and 3E) and flat (Figure 3F) were observed in this assay.

### Myosin Va mRNA expression in different tissues

**Figure 4:**
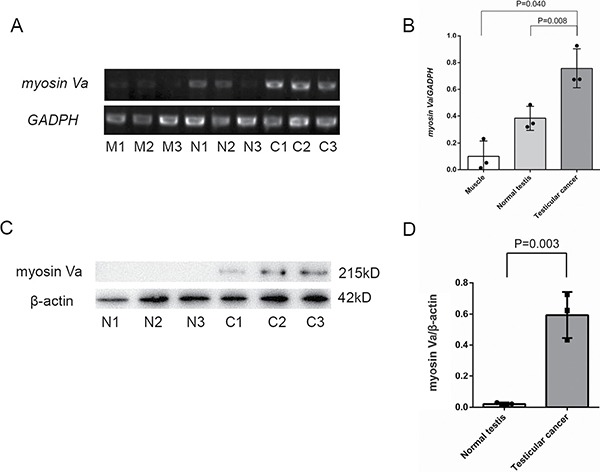
Histological expression level examination of Myosin Va in testicular cancer tissue (**A**) Myosin Va is expressed in muscle tissue, normal tissue and testicular cancer tissue. Every tissue is evenly divided into three samples, which is respectively labeled as M1, M2, M3, N1, N2, N3, C1, C2, C3. Three parallel experiments are conducted for each sample and *GADPH* is served as a reference gene. (**B**) The result shows that Myosin Va mRNA is distributed in all three tissues and the order of expression level from high to low is: testicular cancer tissue, normal tissue and muscle tissue.(**C**) Western blot analysis of myosin Va protein expression in different tissues. The normal testis and testicular cancer tissues are extracted and probed with myosin Va polyclonal antibody. β-actin was serves as a reference protein. (**D**) Testicular cancer shows a higher expression of myosin Va protein than normal testis. The results of the column diagram are in accordance with that of RT-PCR.

Semi-quantitative RT-PCR was used to detect *Myosin Va* mRNA expression in muscle tissue, normal testis and testicular tumor. A 602-bp fragment as a part of *myosin Va* cDNA was amplified (Figure 4A, upper panel). A 452-bp *GAPDH* fragment served as a positive control (Figure 4A, lower panel). The result showed that *myosin Va* mRNA was distributed in all tested tissues. The order of expression from high to low is: testicular tumor, normal testis and muscle tissue (Figure 4B). We could get the preliminary conclusion that myosin Va had a higher transcription level in testicular tissue than normal tissue.

**Figure 5:**
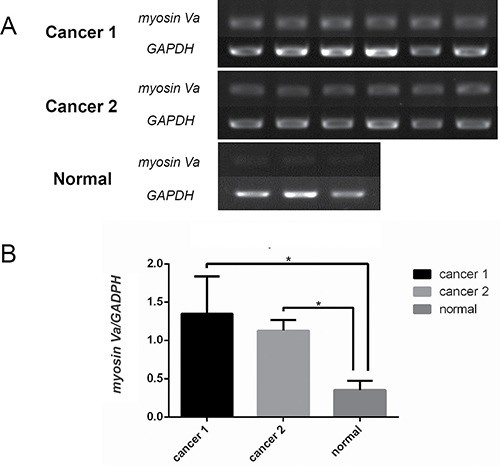
Characterization of myosin Va mRNA expression in prostate cancer Myosin Va's mRNA levels in the samples of two prostate cancer patients and a non-cancer patient are assessed by sqRT-PCR. *GAPDH* is used as reference gene. The results show that myosin Va's mRNA level is higher in prostate cancer tissues than normal tissues.

Similarly, the myosin Va's mRNA expression was also detected in the tissues from two prostate cancer patients and a non-cancer patient (Figure 5A). The results showed that myosin Va's mRNA level was significantly higher in prostate cancer tissues than normal tissues (Figure 5B).

### Identification of myosin Va proteins in normal testis and testicular tumor

Western blot was performed to determine whether myosin Va protein was expressed in normal testis and testicular tumor. The polyclonal antibody recognized a 215-kD band of myosin Va (Figure 4C, upper panel). β-actin served as the positive control (Figure 4C, lower panel). Testicular tumor showed a higher expression of myosin Va protein than normal testis (Figure 4D). Column diagram clearly illustrated the myosin Va's expression level in two tissues, this result was in accordance with that of RT-PCR.

### Localization of myosin Va in normal and tumorous spermatocytes

**Figure 6:**
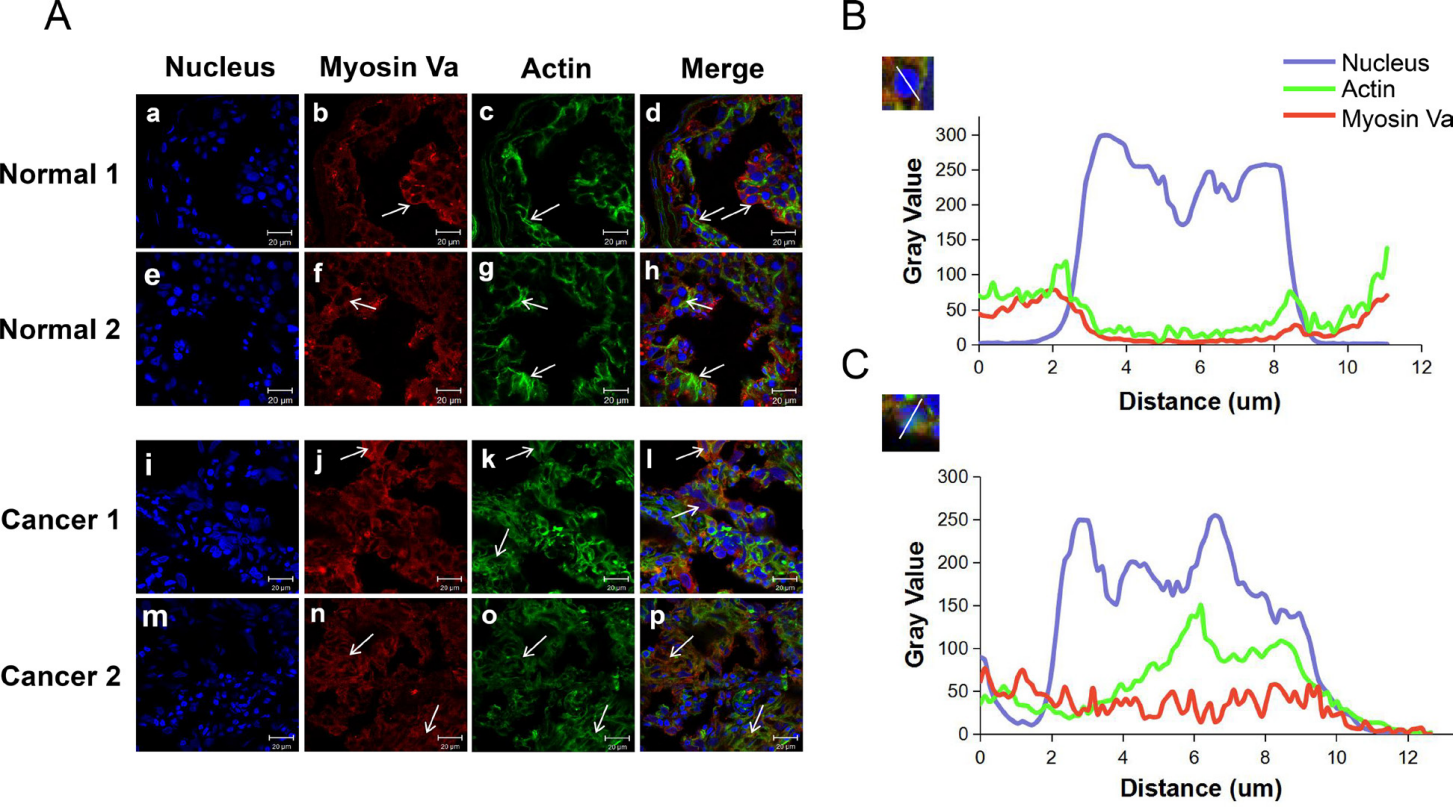
Immunofluorescent localization of myosin Va and actin in normal testis and testicular tumors (**A**) Triple staining at different tissues (blue DAPI nuclear staining, green actin staining with Actin-Tracker Green, red anti-myosin Va antibody). a-h, In normal testes tissue, myosin Va and actin show remarkably co-localization in the periphery of the cell nucleus. Actin presents the obvious fibrous distribution, and myosin Va densely clusters in the actin-abundant region. i-p, In testicular tumor tissue, although myosin Va still co-localizes with actin, myosin Va and actin are diffusely distributed in the whole cell, especially they don't tend to be distributed around the nucleus. Actin shows a dispersed distribution instead of a fibrous structure. The arrows indicate the distribution features of myosin Va and actin. Scale bar, 20 μm. (**B**) This diagram displays the distribution between nucleus, myosin Va and actin. We chose a typical nucleus structure and compared the gray value of the triple staining. The results show that in normal testes, myosin Va and actin are co-localized and distributed in the periphery of the cell nucleus. (**C**) This diagram displays the distribution between the three stains in testicular tumor tissues. Different from the normal tissue showed in Diagram B, myosin Va and actin are diffusely located throughout the cell, instead of being arranged around the nucleus. The distinct distribution patterns of myosin Va and actin between normal and tumor tissue suggest that they may take part in cell tumorigenesis and tumor progression.

Immunofluorescent staining was conducted to localize myosin Va and F-actin in normal testes and testicular tumors. In normal testis tissue, myosin Va and actin were co-localized in the periphery of the cell nucleus (Figure 6A, Normal 1 and 2). Actin-based microfilament represented obvious fibrous distribution (Figure 6A, c and g), and myosin Va densely clustered in the actin-abundant region (Figure 6A, b and f). However, in the testicular tumor tissue, myosin Va and F-actin were diffusely distributed throughout the whole cell (Figure 6A, Cancer 1 and 2). They particularly were not distributed around the nucleus (Figure 6C). The fibrous structure of actin-based microfilament was replaced by its dispersion structure (Figure 6A, k and o), and myosin Va was co-localized with F-actin (Figure 6A, j and n). The different distribution patterns of myosin Va and actin-based microfilament between normal and tumor tissue suggest its functional role in tumor progression.

Typical cell images were chose and the gray value of myosin Va, actin and nucleus dyeing were analyzed. In normal tissue, the higher the nucleus staining gray value was, the lower the myosin Va and actin's staining gray values were, so myosin Va and actin were co-localized in the periphery of the cell nucleus. However, in the tumor tissue, the gray values of three dyeing displayed a random model, which showed that myosin Va and actin were randomly distributed in the whole cell. Another interesting finding was that the gray level of myosin Va in tumor cell nucleus (Figure 6C) was far higher than that in normal cell nucleus (Figure 6B), which meant in normal cells, myosin Va mainly distributed in cytoplasm, but in cancer cells, it diffused into the nucleus and might exert special functions related to tumorigenesis. These phenomena coincided with the hypothesis of ‘chromomyosin’ which we had already put forward in our former review [11].

### The localization of myosin Va in prostate cancer and normal tissues

**Figure 7:**
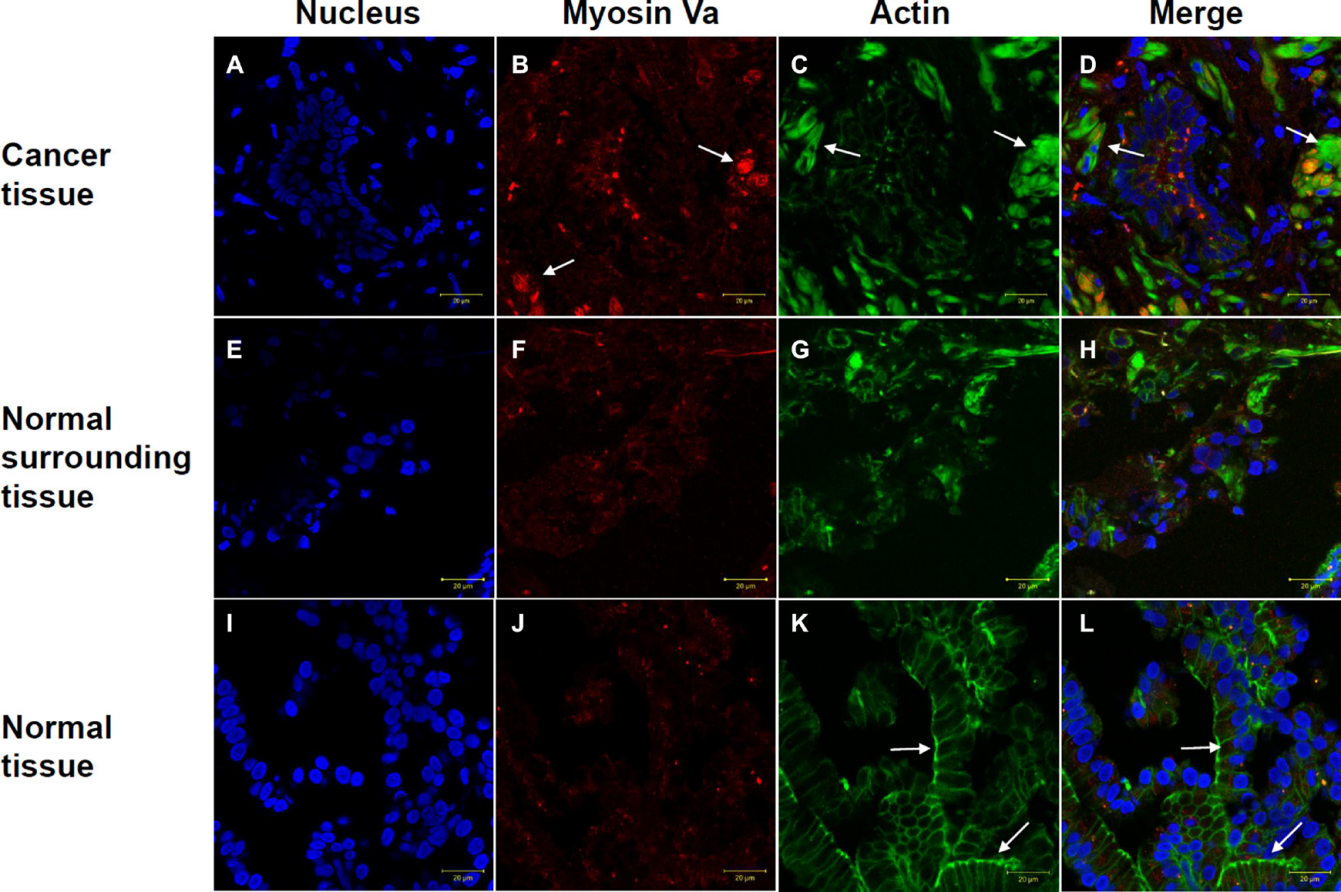
Immunofluorescence staining of myosin Va (red) and F-actin (green) in prostate cancer tissues and normal tissues In prostate cancer tissues, myosin Va has a higher expression level and shows a bulk distribution (**B, F** and **J**). In cancer patient tissues, F-actin presents a diffuse distribution instead of a fibrous structure (**C, G**). In normal tissues, F-actin shows a regular fibrous structure (**K**). Arrows indicate myosin Va and F-actin's specific expressions in different tissues. The arrows in (**B**) show myosin Va's high expression, the arrows in (**C, D**) display F-actin's diffuse distribution in tumor tissues, and the arrows in (**K, L**) demonstrate the obvious fibrous structure of F-actin in normal tissues. Normal surrounding tissues from prostate cancer patient present the transitional characteristics of both cancer tissues and normal tissues (**H**) DAPI (blue), nucleus (**A, E** and **I**). Scale bar, 20 μm.

Immunofluorescence staining was used to assess localization of myosin Va and F-actin in prostate cancer tissues and normal tissues. The nuclei were stained with DAPI (Figures 7A, 7E and 7I). In prostate cancer tissues, myosin Va had a higher fluorescence level and showed a dense bulk distribution (Figures 7B, 7F and 7J). F-actin in tissues from cancer patients presented a diffuse distribution instead of a fibrous one (Figure 7C and 7G). In normal tissues from non-cancer patients, F-actin showed a regular fibrous structure and the cell structure could be clearly observed (Figure 7K). Normal surrounding tissues from prostate cancer patient presented the transitional characteristics of both cancer tissues and normal tissues. Combined with the results from testicular and prostate tissues (Figure 7H), we found myosin Va displayed a higher expression level in both testicular tumor and prostate tumor than normal tissues (Figures 4 and 5). Additionally, in normal tissue, myosin Va co-localized with actin-based microfilament and showed obvious fibrous structure (Figure 7L). However, in tumor tissue, F-actin showed diffuse distribution throughout the whole cell (Figure 7D). As a result, the common functions for myosin Va in the above two cancers were regulating normal microfilament and cell morphology, and promoting actin assembly.

### The roles of myosin Va in the maintenance of normal cell mitosis

**Figure 8:**
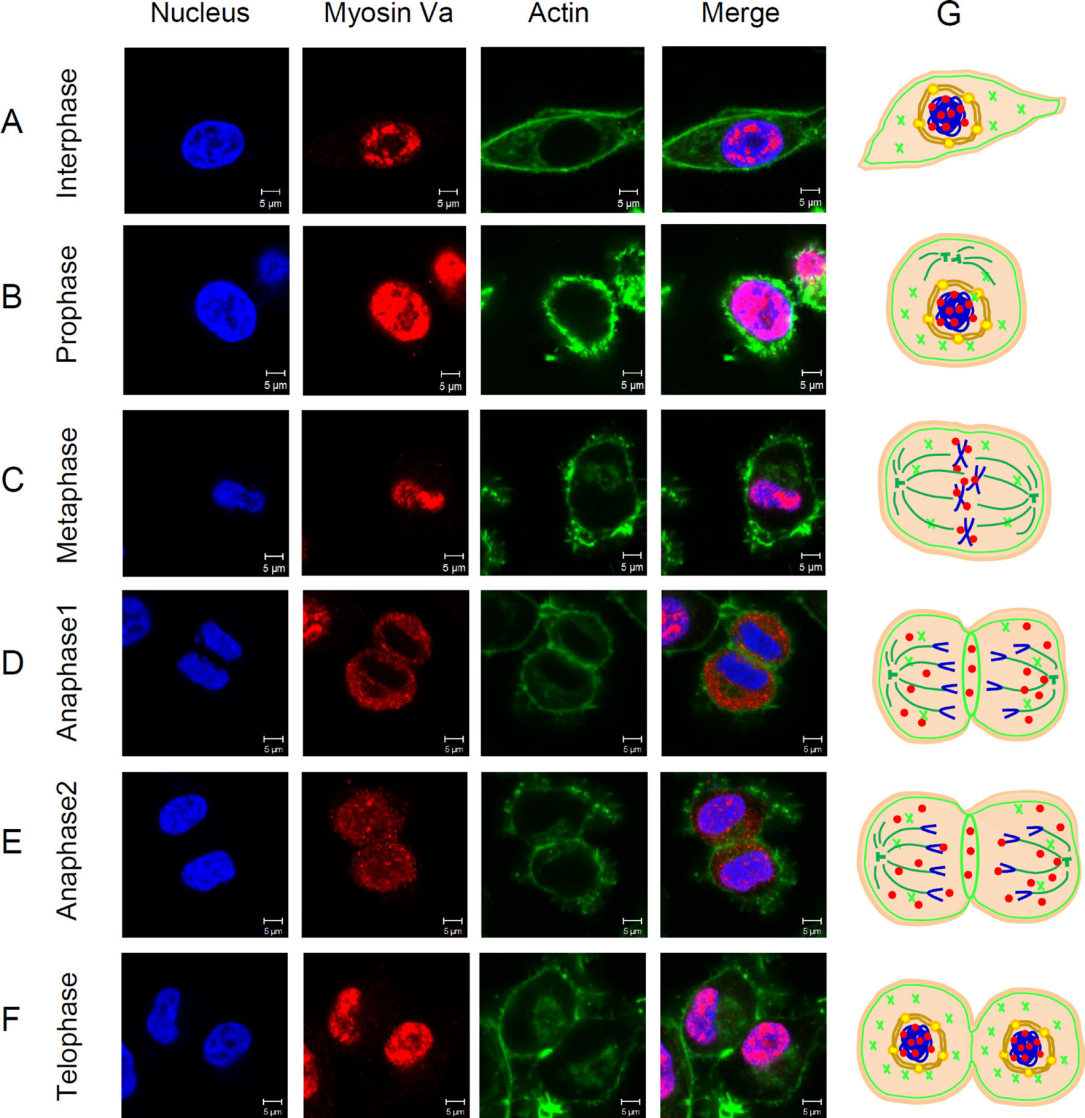
Distribution of myosin Va in cells during mitosis (**A**–**F**) Localization of myosin Va during each phase of the cell cycle is showed by immunofluorescence images. HeLa cells are immunostained using anti-myosin Va antibody and Actin-Tracker Green. Nucleus was stained with DAPI (blue). Scale bar, 5 μm. Myosin Va shows a remarkably co-localization with nucleus during the interphase and prophase of cell mitosis, but distributes in the cytoplasm in the late anaphase, which suggest the dynamic and changeable interactions between myosin Va and nucleus, and these interplays may play an essential role during tumorigenesis. In addition, during the late anaphase, myosin Va also shows a notable co-localization with actin-based microfilament, indicating that myosin Va connects with actin filament and moves along it in this stage, but how myosin Va translocates in and out of nucleus during different mitosis phases still remains unclear. (**G**) Models about how myosin Va, actin and tubulin work duing cell mitosis have been established. In interphase cells, myosin Va is mainly distributed in the nuclear region. In interphase cells, a part of F-actin iss evenly distributed in the cytoplasm, and another part is densely concentrated inside the cell membrane. Myosin Va still co-localizes with nucleus. At prophase, cells gradually turn round and show spherical, and F-actin presents ring-like structure and surrounds the nucleus. At metaphase, the chromosome concentrates on the equatorial plate, myosin Va displays strong co-localization with nucleus. However, after metaphase, this kind of co-localization is weakened and the signals of myosin Va gather in the cytoplasm. At the late anaphase, as the sister chromatids move away, myosin Va is gradually distributed throughout the whole cell, and eventually re-concentrates in the nucleus region. In addition, at anaphase and telophase, F-actin forms a contractile ring separating the dividing cells, and myosin Va is also located at the structure.

To investigate the essential roles of myosin Va during cell mitosis, a series of immunofluorescence images of cells in each phase of the cell cycle were analyzed. In interphase cells, myosin Va was mainly distributed in the nuclear region (Figure 8A), which was similar to the IF results in testicular tumor tissues (Figure 6). The strong co-localization between myosin Va and nucleus in tumor cells implied the special functions of myosin Va during tumorigenesis, and these phenomena were in accordance with the concept of ‘chromomyosin’ presented in our former review [11]. In interphase cells, a part of F-actin was evenly distributed in the cytoplasm, and another part was densely concentrated inside the cell membrane (Figure 8A). The actin cortex located at the interface between the cell and its environment, played a crucial role during cell rounding against a deformable constraint [34]. The actin cortex also enabled cells to sense and respond to force anisotropies [35]. At prophase, Hela cells gradually turned around and showed spherical (Figure 8B), which was essential for effective chromosome capture and spindle stability [36]. At this phase, myosin Va was still co-localized with nucleus, and actin presented ring-like structure surrounding the nucleus (Figure 8B). At metaphase, the chromosome concentrated on the equatorial plate, myosin Va displayed strong co-localization with nucleus (Figure 8C). However, after metaphase, this kind of co-localization was weakened and the signals of myosin Va gathered in the cytoplasm (Figure 8D). During this process, chromosomes moved toward spindle poles by shortening spindle fibers, and myosin Va might take part in the shortening of spindle fibers and providing the shortening force. The poleward chromosome movement was associated with disassembly of the microtubule fibers which linked chromosomes to spindle poles [37]. Now we can conclude that myosin Va is implicated in the disassembly of spindle microtubule and movement of chromosomes.

At the late anaphase, as the sister chromatids moved apart, myosin Va was gradually distributed throughout the whole cell (Figure 8E), and eventually re-concentrated in the nucleus region (Figure 8F). The different localization of myosin Va in different phases suggested its essential functions during cell mitosis. We believed myosin Va worked as chromomyosin, increasing or decreasing expression level in nucleus region during different mitosis phases and regulating karyokinesis. In addition, at anaphase and telophase, actin formed a contractile ring separating the dividing cells, and myosin Va was also located at the region (Figure 8D, 8E and 8F). Myosin Va might participate in the formation of contractile ring and promote cytokinesis. Models showing how myosin Va, actin and tubulin work during cell mitosis have been established (Figure 8G).

**Figure 9:**
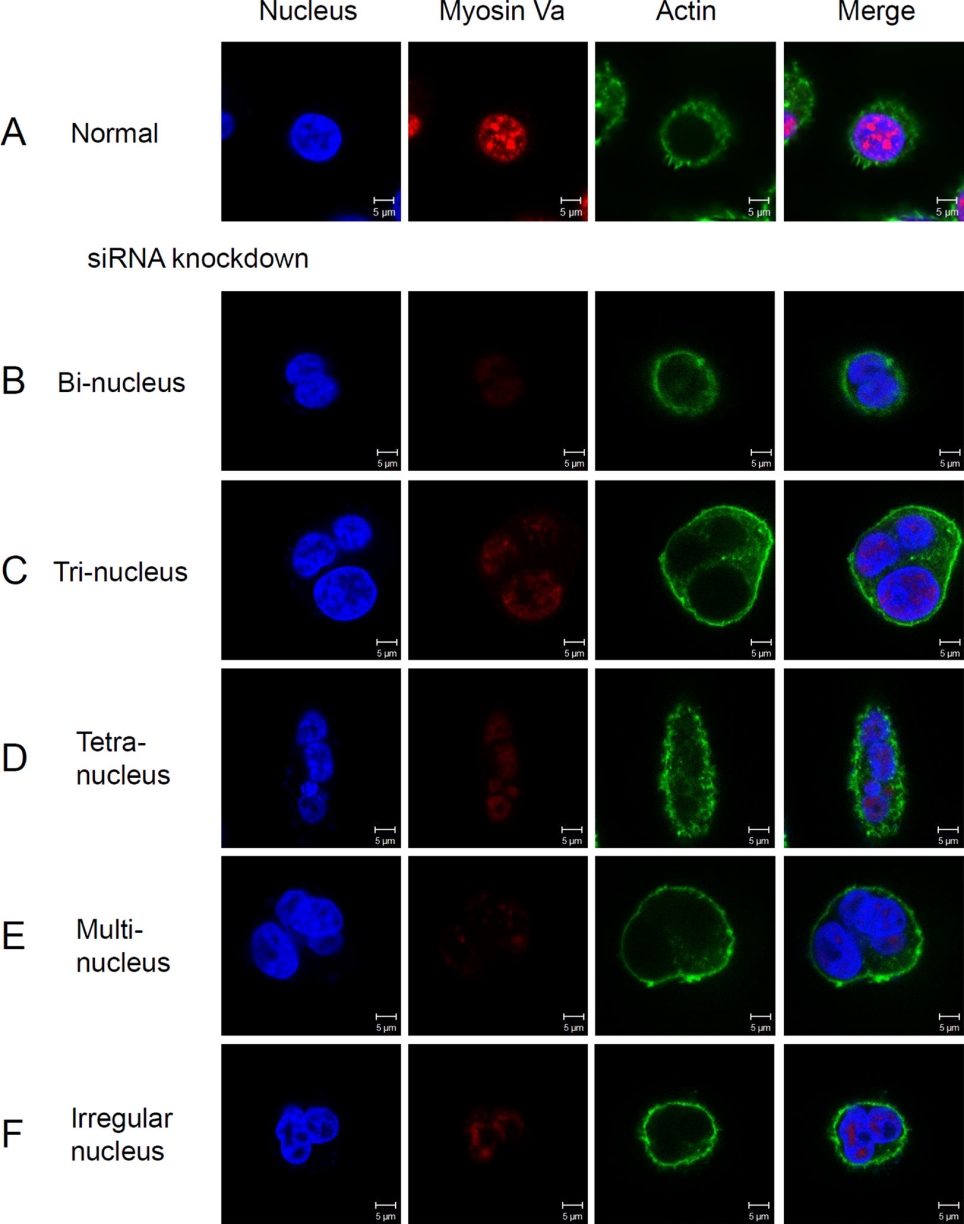
The knock-down of myosin Va prevents normal mitosis and leads to multinucleation Immunofluorescence staining images of normal cell (**A**) and the cells with myosin Va knockdown (**B**–**F**). The siRNA treated cells show a weaker myosin Va fluorescence than normal cancer cells, and they also present different nucleus numbers and forms, including two nuclei (B), three nuclei (C), four nuclei (D), more nuclei (E) and irregular nuclei (F). Nuclei were stained with DAPI (blue). These phenomena suggest that myosin Va functions in cytokinesis and karyokinesis, and lack of myosin Va causes abnormal cell division. Scale bar, 5 μm.

To investigate whether knock-down of myosin Va changes the mitosis of normal cells, Hela cells were stained for nucleus, actin and myosin Va (Figure 9). Results demonstrated that myosin Va fluorescence was weaker in siRNA treated cells, contrasting to cancer cells (Figure 9B–9F). Similar to normal cells, the myosin Va co-localized with nucleus. A part of F-actin was densely concentrated inside the cell membrane, and other actin was evenly distributed in the cytoplasm (Figure 9). However, a remarkable phenomenon was noticed. After siRNA knockdown, most of the cells presented multinucleation. The siRNA treated cells displayed different nucleus number and morphology, including two nuclei (Figure 9B), three nuclei (Figure 9C), four nuclei (Figure 9D), more nuclei (Figure 9E) and irregular nuclei (Figure 9F), which indicated aberrant cell division. In conclusion, these data provided the evidence that myosin Va could facilitate cells to sustain normal cell mitosis.

### Myosin Va and tumor cell migration

Increasing number of research showed the relationship of myosin Va in cell migration, and tumor invasion. In the development of neurons, GFP-M5-overexpressed cells (overexpressing a GFP-tagged head domain of myosin Va) show a slow spreading ability but normal cell migration and lamellipodial dynamics. In contrast, the GFP-M5Δ cells (truncating the tail domain of myosin Va) display a rapid spreading ability but limited cell migration and reduced lamellipodial dynamics [38]. Snail, a zinc finger transcriptional factor [28], can bind to an E-box of the myosin Va promoter and activate myosin Va. The upregulation of myosin Va mediated by Snail promotes the invasion of lung carcinoma epithelial cells and their metastasis [29]. In the future we will seek further evidence for the association between myosin Va and cancer cell migration.

**Figure 10:**
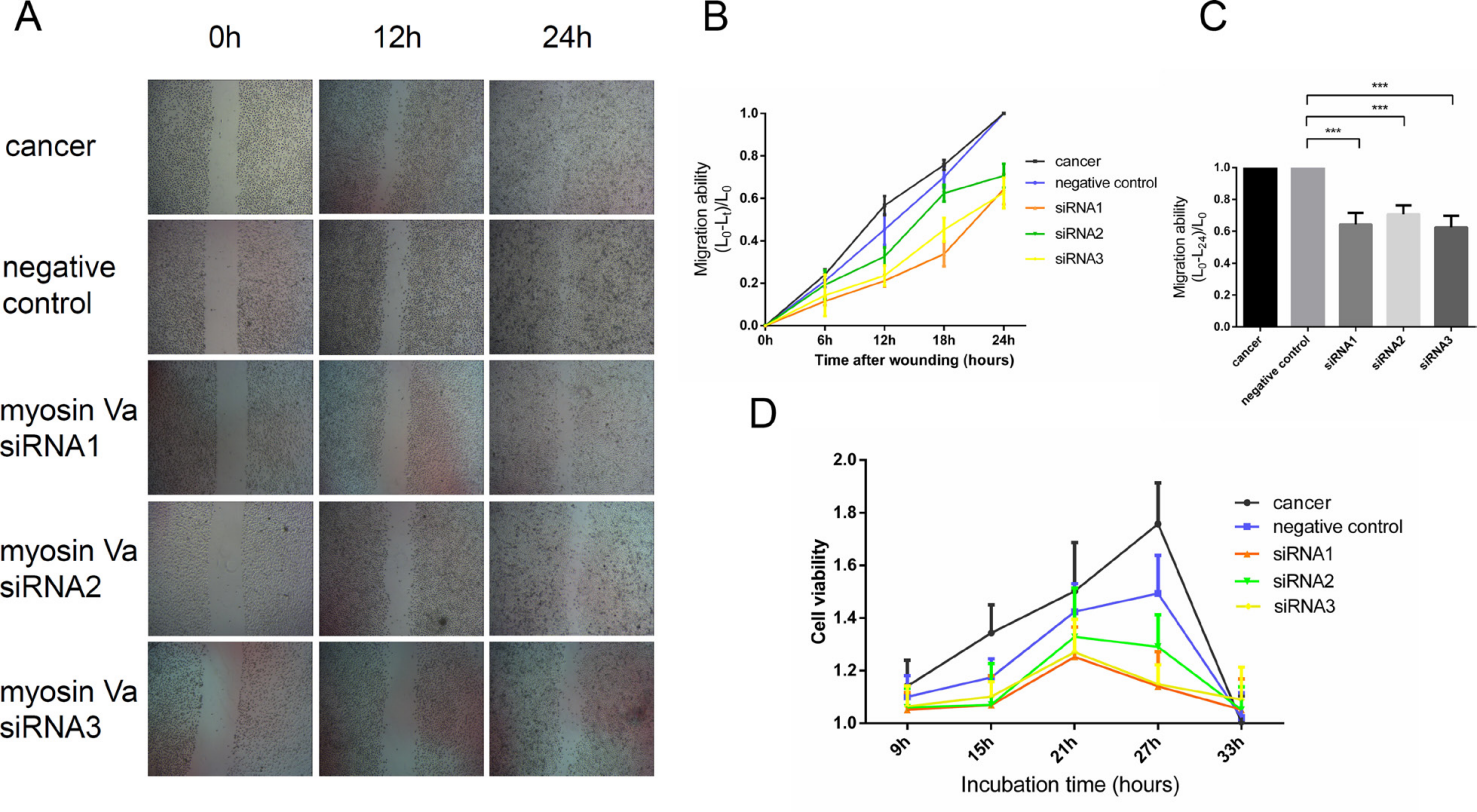
The relationship between myosin Va and tumor cell migration and proliferation (**A**) Cell migration ability is examined by a Wounding Assay. The wounding assay shows that all siRNA treated Hela cells migrate at decreased rates compared to the cancer group (normal Hela cell line) after the incision was made, and these cell lines displays a slower spreading behavior and weaker migration ability. Consistent with RT-PCR results, the effects of siRNA1 and siRNA3 are more significant than those of siRNA2 (Figure 5B and 5C). The negative control group is designed to avoid the disturbance of transfection processes. Wound closure is examined by phase-contrast microscopy at the time of scratching and after 6, 12, 18 and 24 hours. Magnification, ×40. (**B**) This diagram shows the relationship between the time after wounding and tumor cell migration ability. The measurable indicator is the distance to the incision. After the incision was made, the tumor cells would gradually migrate and invade, and as a result, the distance of incision would accordingly shorten. Lt represents the distance of incision at t hours, and at different hours, the value (L0-Lt)/L0 represents the migration ability of the tumor cells. The results show that the Hela cells between the tumor group and negative control group have a similar migration rate, and all siRNA treated Hela cells migrate at decreased rates. (**C**) The diagram displays the association between different cell lines and migration ability. The value (L0-L24)/L0 presents the gross migration ability. The result is in accordance with diagram B (*P* < 0.001). (**D**) This diagram demonstrates the myosin Va function during tumor cell proliferation. CCK-8-based cell counting assays was performed to evaluate cell viability and proliferation ability after 9, 15, 21, 27 and 33 hours of incubation. The Hela tumor cells showed the highest viability and fastest proliferation speed, and the negative control group took second place. Three sets of siRNA treated cells had a slower proliferation speed. Wounding assay and cell counting assay suggest that myosin Va efficiently enhance tumor cell motility and viability.

We used three sets of siRNA targeting myosin Va, siRNA1, siRNA2 and siRNA3, to repress myosin Va expression in Hela cells. The changes of tumor cell migratory ability were analyzed by a wounding assay. As showed by RT-PCR experiment, all siRNAs effectively reduced myosin Va mRNA levels in Hela cells (Supplementary Figure 1), and siRNA1 and siRNA3 showed a more striking interfering efficiency than siRNA2. The wounding assay showed that all siRNA treated Hela cells migrated at a reduced rate after the incision was made, displaying slower spreading behavior. Consistently, the effects of siRNA1 and siRNA3 were more significant than those of siRNA2 (Figure 10A, 10B and 10C). To avoid the disturbance of the transfection process, a negative control group served here for contrasting evidence. The wounding assay showed that Hela cells between tumor group and negative control group had a similar migration rate. As a result, transfection process had almost no effect on the spreading of tumor cells. These results indicate that myosin Va is required for the spread and migration of cancer cells.

### Myosin Va and tumor cell proliferation

Cell Counting kit-8 (CCK-8) assay was performed to evaluate tumor cell proliferation and viability [39]. In the presence of electron-coupling reagents, WST-8 could be reduced to an orange-yellow formazan by some dehydrogenase in the mitochondria. The faster the cells proliferated, the deeper the cell medium color was. As a result, to the same cells, the medium color and the cell number displayed linear relationship [39]. Three sets of siRNA targeting myosin Va, siRNA1, siRNA2 and siRNA3, were used to display the myosin Va-deficient cells viability. The tumor group and negative control served as a contrast. All three knockdowns had a fairly high efficiency (*P* < 0.05) (Supplementary Figure 1). The Hela tumor cells showed the highest viability and fastest proliferation speed, and the negative control group took second place (Figure 10D). The myosin Va-knock down tumor cells had a slower proliferation speed. Consistent with the wounding assay, the siRNA1 and siRNA3 group had a more striking interfering efficiency than siRNA2 and showed lowest viability (Figure 10D). After 27 h of incubation, the cell viability of tumor and negative control groups obviously decreased, and the same things happened to three siRNA sets after 21 h, which may be attributed to the medium nutrient consumption. CCK-8 assay showed a strong relationship between myosin Va knock down and the inhibition of tumor cell proliferation. As a result, after decreasing the myosin Va's expression in Hela cells, the tumor cells’ motility and viability were significantly inhibited (Figure 10). The invasive migration and unlimited proliferation are the hallmarks of malignant tumor cells. As a result, we believe myosin Va's roles imply new oncodiagnosis and -therapies approaches.

## DISCUSSION

To our knowledge, this is the first study that analyzed and compared the expression of myosin Va in testicular tumors, prostate tumors as well as its prognostic significance in patients with the two cancers. Our results showed a strong positive relationship between the myosin Va expression level and testicular/prostate tumorigenesis. In addition, Hela cell line is used to analyze the specific functions of myosin Va during tumor progression, including abnormal cell mitosis, tumor cell proliferation and migration. These also confirm the earlier suggestions that myosin Va might play a role during tumorigenesis.

Our results, showing high mRNA and protein expression of myosin Va in testicular/prostate tumor tissue, are in accordance with previous research, which indicated that myosin Va had a high expression level in colorectal cancer [15] and human lung carcinoma epithelial cells [29]. In addition, at least six other classes of the myosin superfamily also showed similar characteristics: myosin I [11, 40], II [41, 42], VI [43], VII [44], IX [45] and X [46]. The upregulation of myosin Va in tumor tissue will be important to determine which roles myosin Va may play during tumorigenesis.

Tumorigenesis is a complex process which involves genetic unstable transformation and tumor metastasis [11]. During tumor metastasis, malignant neoplastic cells dissociate from the primary tumor and migrate into blood vessels and lymphatic system, and subsequently get flushed and germinate in different tissues or organs [16]. These primary tumor cells alter cell polarity and adhesion, and rapidly form cell protrusions. They invade through the acquisition of cell motility, and induce the degradation of extracellular matrices and basement membranes [47]. Cell proliferation is also the hallmark of tumor progression [42]. These changes are basically related to cellular dynamics and cell motility [48]. As a result, more and more studies began to focus on the roles of the motor protein superfamily during tumorigenesis. Actins for example, provide two kinds of isoforms with opposite functions in tumor cells [49]. β-cytoplasmic actin serves as a tumor suppressor, preventing tumor cell proliferation and migration [50]. In contrast, γ-cytoplasmic actin promotes tumor formation through a series of regulatory proteins, including ERK1/2, WAVE2, p34-Arc, cofilin1, and PP1 [49–52]. Moreover, the ratio of β and γ-actin has been utilized as an oncogenic biomarker at least for lung and colon carcinomas [49].

In the past study, we systematically summarized the diverse and indispensable roles of myosins during tumor formation [11]. As actin-dependent motor proteins translocating along microfilaments by consuming ATP [53], myosins take part in cell migration, karyokinesis and cytokinesis, organelle/cargo trafficking, signal transduction and phagocytosis [54]. Furthermore, myosins regulate cell polarity, cell-cell adhesion, protrusion formation, apoptosis suppression, and tumor metastasis during tumorigenesis [42, 55]. However, most of the research pays close attention to the functions of myosin II and VI [56–58], and there are few studies exploring the specific roles myosin Va in tumor formation. As a result, we wish to elucidate in which of the processes characterizing tumorigenesis myosin Va functions. The exploration of the varied and complex relationships between myosin Va and tumorigenesis will be important for future oncodiagnosis and oncotherapy.

In our study, the distribution patterns of myosin Va and microfilament in normal testes and testicular tumor were analyzed and compared. We found that in normal tissues, myosin Va and actin are co-localized and show a fibrous distribution pattern, and that they are mainly located around the nucleus (Figure 6A and 6B). However, in testicular tumor tissues, although myosin Va and actin are still co-localized, the two protein represent a diffuse distribution throughout the cells (Figure 6C). The transformation of the distribution patterns of myosin Va and actin-based microfilament indicates that they are likely responsible for testicular tumorigenesis.

The roles of actin during cell mitosis have been revealed recently. Almost all cells turn round and are spherical when entering mitosis [59], which is essential for effective chromosome capture and spindle stability [36]. The actin cortex located at the interface between the cell and its environment, plays a crucial role during cell rounding against a deformable constraint [34]. The actin cortex also enables cells to sense and respond to force anisotropies [35]. Furthermore, a ring-like cytoplasmic actin surrounding the spindles gradually forms during the metaphase [60]. Actin further regulates astral microtubule dynamics, centrosome separation [61] and the positioning and orientation of mitotic spindles [62, 63]. The co-localized distribution (Figure 3A) between myosin Va and actin indicates their functional connection. Myosin Va may also participate in cell mitosis.

Previous research showed the indispensable association between myosin Va and cell division. Myosin Va, as a actin-dependent motor protein, functions together with the microtubules [64–66] and microfilaments during mitosis. BDM, serving as a myosin inhibitor (including myosin II, V and VI) [67], effectively disturbed the chromosome movement during anaphase and the completion of cytokinesis, proving that myosins are related to normal cell division [68]. The myosin Va-deficient fibroblasts are twice as likely to form binucleus as wild type fibroblasts, indicating that myosin Va is relevant for efficient cell division [64]. During interphase, myosin Va concentrates on the microtubule organizing center (MTOC), and myosin Va binds to the microtubules either directly or indirectly via microtuble-associated protein [64]. However, in our study, a different distribution of myosin Va presents in the tumor cells. At interphase, prophase, metaphase and telophase, myosin Va mainly concentrates in nucleus region (Figure 8A, 8B, 8C and 8F). The strong co-localization between myosin Va and nucleus in tumor cells imply the myosin Va's special functions during tumorigenesis, which are in accordance with the concept of ‘chromomyosin’ put forward in our former review [11]. At early anaphase, myosin Va is mainly located in cytoplasm, and at late anaphase, myosin Va re-distributes in nucleus region. During anaphase, the spindle fibers shorten and chromosomes moved toward spindle poles, and myosin Va might provide the force to promote the shortening of spindle fibers. The poleward chromosome movement was related to the disassembly of microtubule fibers linking chromosomes to spindle poles [37]. As a result, we can conclude that myosin Va is implicated in the disassembly of spindle microtubule and normal movement of chromosomes.

In a symmetrically dividing cell, the cleavage furrow ingression at the equatorial cortex is driven by the constriction of an actomyosin contractile ring [69], and then the contractile ring progressively squeezes the central spindle and forms the midbody [70]. The midbody offers a platform for recruitment of many proteins mediating final cytokinesis [71, 72]. During cell mitosis, actin forms a contractile ring-related structure separating the dividing cells, and myosin Va is also located at the region (Figure 8D, 8E and 8F). Based on these observations, we believe that myosin Va plays an essential role in the organization of contractile ring and midbody-related structures. Myosin Va interacts with microtubules and actin filaments during karyokinesis and cytokinesis, and promotes an organized progress of cell division. The localization of actin and myosin Va in midbody region indicates that the formation of the midbody might rely on: 1) the assembly of central spindles which are regulated by myosin Va; 2) and certain proteins or molecules transported by the myosin Va cargo binding domain.

We put forward a new concept of “chromomyosin” in our previous paper [11], which means an unconventional myosin existing in nuclear matrix and interacting with nuclear monomer actin. In this study, we find that the expression level of myosin Va in tumor cell nucleus (Figure 6C) is significantly higher than that in normal cell nucleus (Figure 6B). In normal cells, myosin Va mainly distributes in cytoplasm, but in cancer cells, it diffuses into the nucleus and may exert special functions related to tumorigenesis. In addition, the cell immunofluorescence images in each phase of the cell cycle show that in interphase, prophase, metaphase and telophase, the myosin Va is mainly distributed in the nuclear region (Figure 8). The strong co-localization between myosin Va and nucleus in tumor cells implies the special functions of myosin Va during tumorigenesis, and these phenomena are in accordance with the concept of ‘chromomyosin’. However, in anaphase, the co-localization is weakened and the signals of myosin Va gathers in the cytoplasm (Figure 8D). During this process, chromosomes move toward spindle poles by shortening spindle fibers and the disassembly of the microtubule fibers which linked chromosomes to spindle poles [34]. Myosin Va might take part in providing the shortening force, the disassembly of spindle microtubule and movement of chromosomes. The different localization of myosin Va in different mitosis phases suggest its indispensable roles in karyokinesis and cytokinesis.

In this research, we use siRNA knock-down to explore the specific detailed functions of myosin Va during tumor cell mitosis. Hela cells are utilized as experimental models. Overexpressed myosin Va may enhance actin bundling by crosslinking actin filaments. Additionally, myosin Va may also promote actin bundle formation by crosslinking actin filaments and tubulin microtubules [73–75]. Although myosin Va overexpression induces actin bundles, myosin Va is not indispensable for actin formation. Myosin Va knock-down assays show that siRNA treated cells present aberrant nucleus number and morphology, including two nuclei (Figure 9B), three nuclei (Figure 9C), four nuclei (Figure 9D), more nuclei (Figure 9E) and irregular nuclei (Figure 9F). These multi-nucleation and aberrant nucleation indicate that myosin Va plays an essential roles in maintaining normal mitosis.

Wounding assay and CCK-8 assay were performed to evaluate tumor cell migration and proliferation. The myosin Va knock-down cells all showed a slower migration speed and lower proliferation ability (Figure 10). These results strongly proved that myosin Va is an indispensable motor protein during tumor metastasis. Myosin Va could effectively enhance tumor cell motility and viability. Opposite to myosin Va, myosin Vb is another member of the class V of unconventional, dimeric nonfilamentous myosins [76], whose inactivation accelerates tumor cell migration and invasion [77]. The mutation of myosin Vb could induce diseases related to microvillus inclusion and disrupt epithelial cell polarity [78]. A loss of cell polarity is commonly regarded as the hallmark of cancers, which promote tumor cells to invade into adjacent regions and activate tumor metastasis [79]. Dong et al (2013) showed that myosin Vb is epigenetically silenced in gastric tumor cells because of aberrant DNA methylation and histone modification [80]; the downregulation of myosin Vb may lead to gastric tumorigenesis [77]. As a result, myosin Va plays a opposite role to myosin Vb during tumorigenesis. In tumor cells, myosin Va has a higher expression level than normal cells. When myosin Vb is down-regulated; the inactivation of myosin Va may inhibit proliferation, invasion, and motility of tumor cells, whereas myosin Vb could promote these activities. The antagonistic effects between myosin Va and Vb may regulate the physiological processes of tumor cells.

In conclusion, our experiments clearly demonstrated an overexpression of myosin Va in testicular tumors and its functions during tumorigenesis. These functions contain promoting rapid cell division, stabilizing cell-cell adhesion, as well as enhancing tumor migration and proliferation. How exactly myosin Va interacts with actin, tubulin and other regulatory proteins remains a challenging area of investigation. More evidence remains to be found for the involvement of myosin Va that then could be useful for future technologies in tumor diagnosis and anti-tumor treatments.

## MATERIALS AND METHODS

### Tissue, cell lines and culture conditions

Human testes and prostate tissue samples were obtained at the time of surgery at the First Affiliated Hospital of Zhejiang University, Zhejiang, China. All samples were obtained with informed patient consent form and with institutional review board approval of the hospital.

Hela cells were used for exploring the roles of myosin Va in mitosis, tumor cell migration and proliferation. Hela cell lines (CCL-2) were cultured in DMEM supplemented with 10% fetal bovine serum, at 37°C in an atmosphere containing 5% CO2.

### HE staining (hematoxylin-eosin staining)

Testicular cancer and prostate cancer tissue samples were fixed in 10% neutral-buffered formalin and embedded in paraffin at room temperature. Histopathologic representative tumor samples were defined on hematoxylin and eosin-stained sections and marked on the slides. Photomicrographs demonstrating the entire section were collected by Olympus BX51 Microscope (Japan).

### RNA extraction and reverse transcription

Total RNA was prepared using the Phase Lock Gel™ Heavy with Trizol A+ (Tiangen Biotech, Beijing, China) from muscle tissue, normal testis and testicular tumor. Every tissue was evenly cut into three samples, and was separately labeled as M1, M2, M3, N1, N2, N3, C1, C2 and C3. On the other hand, the total RNA was prepared with the same method from prostate cancer tissues and normal prostate tissue. Every cancer tissue was evenly cut into six samples and normal tissue for three samples. First, the samples were dissolved in the Trizol A+ and homogenized. Then, the homogenates were subsequently transferred in Phase Lock Gel^™^ Heavy with chloroform, isopropanol and 75% ethanol. The precipitated RNA was suspended in diethylpyrocarbonate (DEPC)-H2O and the RNA concentrations were measured by spectrophotometer. The re-suspended RNA was stored at **−**40°C for reverse transcription. The reversed products were conducted using PrimeScript^®^ RT reagent Kit (Takara, Dalian, China) and stored at **−**20°C for future PCR.

### Semi-quantitative real time polymerase chain reaction (sqRT-PCR)

Two specific primers of myosin Va were designed to analyze the *myosin Va* mRNA expression levels in different tissues. Two specific primers of *GAPDH* were designed as the internal control. The sequences for the primers are listed in Table 1. Muscle tissue, normal testis and testicular tumor were from the same patient. Prostate cancer tissues from two different patients were used in the same method separately. Normal prostate tissue was from a non-cancer patient. The amplification procedures were as follows: 94°C for 5 min; 34 cycles of 94°C for 30 s, 55°C for 30 s, and 72°C for 30 s; 72°C for 10 min for the final extension. The RT-PCR products were visualized by agar gel electrophoresis. The results were analyzed with ImageJ2× and GraphPad Prism 5 software.

**Table 1:** PCR primer sequences employed in sqRT-PCR

Gene name	Sense	Antisense	Size (bp)
A part of *Myosin Va*	5′-ACAGTGGGGCATCAGTTCAG-3′	5′-TACCTCCTGCGGACCACATA-3′	602
*GAPDH*	5′-ACCACAGTCCATGCCATCAC-3′	5′-TCCACCACCCTGTTGCTGTA-3′	452

### Western blot analysis

The normal testes and testicular tumors were homogenized in RAPI Lysis Buffer (50 mM Tris-HCl pH 7.4, 150 mM NaCl, 1% NP-40, 0.1% SDS) with protein inhibitors for 20 min on ice. The lysates were centrifuged at a speed of 14,000 g for 10 min at 4°C. The supernatant were ultrafiltrated by Centricon Centrifugal Filter Unit YM-30 (Millipore) and the protein concentration was measured by spectrophotometer. Then the protein samples were separated on SDS-PAGE with adjusted loading amounts according to their different concentrations. The proteins were then electrophoretically transferred to a PVDF membrane (Bio-Rad, CA, USA). The PVDF membrane was blocked in 5% skimmed milk in PBST (PBS with 0.02 % Tween 20) for 1.5 h and then incubated with rabbit anti-myosin Va antibody (diluted 1:500) and mouse anti-β actin antibody (diluted 1:1000) at 4°C overnight (The antibodies’ information is listed in Table 2). Then, after washing 3 times in PBST for each period of 15 min, the PVDF membrane was incubated in secondary antibody HRP-conjugated goat anti-rabbit IgG (diluted 1:1000) and HRP-conjugated goat anti-mouse IgG (diluted 1:1000) for 1 h. After washing another 3 times for each 15 min, Immunoblots were developed using Pierce ECL Western Blotting Substrate (Thermo).

**Table 2:** Information regarding antibodies employed in Western blot and immunofluorescence

Name	Vender	Cat. no.	Species	Dilution	Purpose
Myosin Va	Sangon Biotech	D153174	Rabbit polyclonal IgG	1:500	Western blot and immunofluorescence
β-actin	Beyotime	AA128	Mouse monoclonal IgG	1:1000	Western blot
Anti-rabbit	Beyotime	A0208	Goat polyclonal IgG	1:1000	Western blot (secondary antibody)
Anti-mouse	Beyotime	A0216	Goat polyclonal IgG	1:1000	Western blot (secondary antibody)

### Immunofluorescent staining at tissue level

The normal testes and testicular tumors were fixed in 4 % PFA (pH 7.4) overnight, and after being washed for 3 times (5 min each time) by PBS, the tissues were dehydrated in 0.5 M sucrose in PBS overnight. The tissues were then embedded in Tissue-Tek O.C.T tissue freezing medium and sliced to 5-μm frozen sections and stored at **−**40°C. Three sections, numbered 1-3, were prepared for detecting the localization of myosin Va and actin in testicular tumors (1) and normal testes (2), with the third serving as a control. The prepared samples were contacted with PBS-Triton (PBS with 0.1 % Triton X-100) for 15 min at room temperature and 1 % bovine serum albumin (BSA) in PBS-Triton was used for 1 h at room temperature to block unspecific regions. Subsequently, section 1 and 2 were incubated in rabbit anti-myosin Va antibodies (diluted 1:500), and section 3 was steeped in PBST. Each incubation was conducted overnight. Then, after washing in PBST 3 times for 10 min each, the sections were incubated in secondary antibodies or dyes for 1 h in the dark. Sections 1 and 2 were incubated in Alexa Fluor 555-labeled goat anti-rabbit IgG (1:1000 dilution, Beyotime, Shanghai). β-Actin was detected by Actin-Tracker Green (1:400 dilution, Beyotime, Shanghai) and nuclei were stained with 4, 6-diamidino-2-phenylindole (DAPI) (Beyotime, Shanghai) contained in the Antifade Mounting Medium (Vectashield, Vector Laboratories). The intracellular localization of myosin Va, actin, and nucleus were observed with a Confocal Laser-scanning Microscope (CLSM510; Carl Zeiss, Germany) fit with appropriate filters and images captured with an Orca II CCD camera (Hamamatsu, Bridgewater, NJ). For controls, the primary antibody was omitted (data not shown).

The prostate cancer tissues, normal surrounding tissues and normal prostate tissues were treated as the same method. The primary antibody is rabbit anti-myosin Va antibodies (diluted 1:500), and the secondary antibody is Alexa Fluor 555-labeled goat anti-rabbit IgG (1:1000 dilution, Beyotime Shanghai). β-Actin was detected by Actin-Tracker Green (1:400 dilution, Beyotime Shanghai) and nuclei were stained with DAPI (Beyotime, Shanghai).

### Myosin Va knockdown by siRNA

Three specific sequences of small interfering RNA (siRNA) targeting different regions of human myosin Va mRNA sequence were designed, and a negative control siRNA was designed for contrast. The detailed information of siRNA was listed in Table 3. The siRNAs were transfected into Hela cells after 24 hours of culture. Lipofectamine^®^ 3000 transfection reagent (Invitrogen, Guangzhou, China) were used for siRNA transfection. In 24-well culture dishes, lipofectamine in transfection reagent (0.75 μl) was added to 50 μl Opti-MEM serum-free medium containing 10 ng/μl of each siRNA oligo, incubated for 5 minutes, and added to the dishes containing 500 μl medium. After 48 hours, the efficacy of myosin Va silencing (the reduction of gene expression) was determined. Then the myosin Va-knockdown Hela cells were detected by immunofluorescent staining at cell level to study the detailed mechanism of action of myosin Va during cell mitosis.

**Table 3:** Primer sequences employed in knock-down

Oliga DNA sequence		Sense	Antisense
siRNA1	Oligo-1, 2	5′-GATCACTAATACGACTCACTAT AGGGGTATAGTCCTAGTAGCTATTT-3′	5′-AAATAGCTACTAGGACTATACCCCT ATAGTGAGTCGTATTAGTGATC-3′
	Oligo-3, 4	5′-AAGTATAGTCCTAGTAGCTATC CCTATAGTGAGTCGTATTAGTGATC-3′	5′-GATCACTAATACGACTCACTATA GGGATAGCTACTAGGACTATACTT-3′
siRNA2	Oligo-1, 2	5′-GATCACTAATACGACTCACTAT AGGGCAGCCGTTTTGGGAAGTATTT-3′	5′-AAATACTTCCCAAAACGGCTGCC CTATAGTGAGTCGTATTAGTGATC-3′
	Oligo-3, 4	5′-AACAGCCGTTTTGGGAAGTAT CCCTATAGTGAGTCGTATTAGTGATC-3′	5′-GATCACTAATACGACTCACTATA GGGATACTTCCCAAAACGGCTGTT-3′
siRNA3	Oligo-1, 2	5′-GATCACTAATACGACTCACTAT AGGGGGATAAGACGGTCCGTAAATT-3′	5′-AATTTACGGACCGTCTTATCCCC CTATAGTGAGTCGTATTAGTGATC-3′
	Oligo-3, 4	5′-AAGGATAAGACGGTCCGTAAA CCCTATAGTGAGTCGTATTAGTGATC-3′	5′-GATCACTAATACGACTCACTAT AGGGTTTACGGACCGTCTTATCCTT-3′
Negative control	n-Oligo-1, 2	5′-GATCACTAATACGACTCACTATA GGGGGGATGTCTCACATCTTGTTT-3′	5′-AAACAAGATGTGAGACATC CCCCCTATAGTGAGTCGTATTAGTGATC-3′
	n-Oligo-3, 4	5′-AAACAAGATGTGAGACATCCCC CCTATAGTGAGTCGTATTAGTGATC-3′	5′-GATCACTAATACGACTCACTATAG GGACAAGATGTGAGACATCCCTT-3′

### Immunofluorescent staining at the cell level

Hela cells were seeded upon cover slips at the bottom of culture dishes and were then fixed with 4% paraformaldehyde for 15 min. 0.25% Triton X-100 was used to incubate cells for 10 min and then PBS was used to wash cells. To block nonspecific reactions, PBST (with 1% BSA) was added for 30 minutes, and then rabbit anti-myosin Va antibodies (diluted 1:500) were added. Each incubation was conducted overnight. Then, after washing in PBST 3 times for 5 min each, the cells were incubated in secondary antibodies or dyes for 1 h in the dark. β-actin was detected by Actin-Tracker Green (1:400 dilution, Beyotime, Shanghai) and nuclei were stained with DAPI (Beyotime, Shanghai) contained in an Antifade Mounting Medium (Vectashield, Vector Laboratories). Imaging was performed by a Confocal Laser-scanning Microscope (CLSM510; Carl Zeiss, Jena, Germany).

### *In vitro* wound-healing migration assay

Tumor cells were seeded in 12-well culture dishes at a density of 1 × 10^5^ cells per well. 5 groups of experiments were carried out simultaneously. The 5 groups were: no treatment (NT), adding negative control siRNA (NC), adding siRNA1 (S1), adding siRNA2 (S2), and adding siRNA3 (S3). An incision was made after 24 hours in the central region of confluence in the culture dish. Cultures were observed at the time of scratching and after 6, 12, 18 and 24 hours, and pictures were taken of 6 separate fields of the wounded area using a phase-contrast microscope (DPIXEL, Singapore). The distance between two broad edges of tumor cells was measured and analyzed by Leica LAF software and GraphPad Prism 5 software.

### Cell proliferation

Cell viability was assessed by the Cell Counting Kit-8 (CCK-8 kit, Beyotime Biotechnology, Shanghai) [39]. Hela cells were seeded at an initial concentration of 2000/well in a 96-well plate containing DMEM supplemented with 10% FBS. DMEM with FBS and PBS were respectively taken as control groups. CCK-8 assay was performed to evaluate cell viability and proliferation ability after 9, 15, 21, 27 and 33 hours of incubation. Then 10 μl CCK-8 solution was added into the culture medium of each sample, followed by incubation at 37°C for 0.5 h. The optical density value at 450 nm was detected using a microplate reader (Synergy™ H1, BioTek, town, state, USA). Eight parallel experimental groups in each treatment sample were used to assess cell viability.

### Statistical analyses

All data were presented as mean ± SD. A student two tailed *t*-test was used to compare the difference between two groups. *P*-values less than 0.05 were regarded as statistically significant.

## SUPPLEMENTARY MATERIALS FIGURES AND TABLES



## References

[1] Huyghe E, Matsuda T, Thonneau P (2003). Increasing incidence of testicular cancer worldwide: a review. J Urol.

[2] Oosterhuis JW, Looijenga LH (2005). Testicular germ-cell tumours in a broader perspective. Nat Rev Cancer.

[3] Skakkebaek NE (1972). Possible carcinoma-in-situ of the testis. Lancet.

[4] McGlynn KA, Cook MB (2009). Etiologic factors in testicular germ-cell tumors. Future Oncol.

[5] Rajpert-De Meyts E, Jørgensen N, Brøndum-Nielsen K, Müller J, Skakkebaek NE (1998). Developmental arrest of germ cells in the pathogenesis of germ cell neoplasia. APMIS.

[6] Siegel RL, Miller KD, Jemal A (2017). Cancer Statistics, 2017. CA Cancer J Clin.

[7] Ilic D, O’Connor D, Green S, Wilt TJ (2011). Screening for prostate cancer: an updated Cochrane systematic review. BJU Int.

[8] Tewari A, Sooriakumaran P, Bloch DA, Seshadri-Kreaden U, Hebert AE, Wiklund P (2012). Positive surgical margin and perioperative complication rates of primary surgical treatments for prostate cancer: a systematic review and meta-analysis comparing retropubic, laparoscopic, and robotic prostatectomy. Eur Urol.

[9] Lengauer C, Kinzler KW, Vogelstein B (1998). Genetic instabilities in human cancers. Nature.

[10] Sluder G, Nordberg JJ (2004). The good, the bad and the ugly: the practical consequences of centrosome amplification. Curr Opin Cell Biol.

[11] Li YR, Yang WX (2016). Myosins as fundamental components during tumorigenesis: diverse and indispensable. Oncotarget.

[12] Vogelstein B, Kinzler KW (1993). The multistep nature of cancer. Trends Genet.

[13] Friedl P, Bröcker EB (2000). The biology of cell locomotion within three-dimensional extracellular matrix. Cell Mol Life Sci.

[14] Tarin D, Thompson EW, Newgreen DF (2005). The fallacy of epithelial mesenchymal transition in neoplasia. Cancer Res.

[15] González L, Eiró N, González-Reyes S, Andicoechea A, González LO, García-Muñiz JL, Vizoso FJ (2012). Clinical significance of myosin in colorectal cancer. Ann Diagn Pathol.

[16] Friedl P, Wolf K (2003). Tumour-cell invasion and migration: diversity and escape mechanisms. Nat Rev Cancer.

[17] Krendel M, Mooseker MS (2005). Myosins: tails (and heads) of functional diversity. Physiology (Bethesda).

[18] Mooseker MS, Cheney RE (1995). Unconventional myosins. Annu Rev Cell Dev Biol.

[19] Baker JP, Titus MA (1998). Myosins: matching functions with motors. Curr Opin Cell Biol.

[20] Mercer JA, Seperack PK, Strobel MC, Copeland NG, Jenkins NA (1991). Novel myosin heavy chain encoded by murine dilute coat colour locus. Nature.

[21] Espreafico EM, Cheney RE, Matteoli M, Nascimento AA, De Camilli PV, Larson RE, Mooseker MS (1992). Primary structure and cellular localization of chicken brain myosin-V (p190), an unconventional myosin with calmodulin light chains. J Cell Biol.

[22] Cheney RE, O’Shea MK, Heuser JE, Coelho MV, Wolenski JS, Espreafico EM, Forscher P, Larson RE, Mooseker MS (1993). Brain myosin-V is a two-headed unconventional myosin with motor activity. Cell.

[23] Reck-Peterson SL, Provance DW, Mooseker MS, Mercer JA (2000). Class V myosins. Biochim Biophys Acta.

[24] Bement WM, Hasson T, Wirth JA, Cheney RE, Mooseker MS (1994). Identification and overlapping expression of multiple unconventional myosin genes in vertebrate cell types. Proc Natl Acad Sci USA.

[25] Altmann K, Frank M, Neumann D, Jakobs S, Westermann B (2008). The class V myosin motor protein, Myo2, plays a major role in mitochondrial motility in Saccharomyces cerevisiae. J Cell Biol.

[26] Wu XS, Rao K, Zhang H, Wang F, Sellers JR, Matesic LE, Copeland NG, Jenkins NA, Hammer JA (2002). Identification of an organelle receptor for myosin-Va. Nat Cell Biol.

[27] Sun X, He Y, Hou L, Yang WX (2010). Myosin Va participates in acrosomal formation and nuclear morphogenesis during spermatogenesis of Chinese mitten crab Eriocheir sinensis. PLoS One.

[28] Cano A, Pérez-Moreno MA, Rodrigo I, Locascio A, Blanco MJ, MG del Barrio, Portillo F, Nieto MA (2000). The transcription factor snail controls epithelial-mesenchymal transitions by repressing E-cadherin expression. Nat Cell Biol.

[29] Lan L, Han H, Zuo H, Chen Z, Du Y, Zhao W, Gu J, Zhang Z (2010). Upregulation of myosin Va by Snail is involved in cancer cell migration and metastasis. Int J Cancer.

[30] Du YC, Lewis BC, Hanahan D, Varmus H (2007). Assessing tumor progression factors by somatic gene transfer into a mouse model: Bcl-xL promotes islet tumor cell invasion. PLoS Biol.

[31] Williams A, Hayashi T, Wolozny D, Yin B, Su TC, Betenbaugh MJ, Su TP (2016). The non-apoptotic action of Bcl-xL: regulating Ca(2+) signaling and bioenergetics at the ER-mitochondrion interface. J Bioenerg Biomembr.

[32] Baarlink C, Wang H, Grosse R (2013). Nuclear actin network assembly by formins regulates the SRF coactivator. MAL. Science.

[33] Philimonenko VV, Janácek J, Harata M, Hozák P (2010). Transcription-dependent rearrangements of actin and nuclear myosin I in the nucleolus. Histochem Cell Biol.

[34] Lancaster OM, Le Berre M, Dimitracopoulos A, Bonazzi D, Zlotek-Zlotkiewicz E, Picone R, Duke T, Piel M, Baum B (2013). Mitotic rounding alters cell geometry to ensure efficient bipolar spindle formation. Dev Cell.

[35] Lancaster OM, Baum B (2014). Shaping up to divide: coordinating actin and microtubule cytoskeletal remodelling during mitosis. Semin Cell Dev Biol.

[36] Gibson WT, Veldhuis JH, Rubinstein B, Cartwright HN, Perrimon N, Brodland GW, Nagpal R, Gibson MC (2011). Control of the mitotic cleavage plane by local epithelial topology. Cell.

[37] Asbury CL (2017). Anaphase A: Disassembling Microtubules Move Chromosomes toward Spindle Poles. Biology (Basel).

[38] Eppinga RD, Peng IF, Lin JL, Wu CF, Lin JJ (2008). Opposite effects of overexpressed myosin Va or heavy meromyosin Va on vesicle distribution, cytoskeleton organization, and cell motility in nonmuscle cells. Cell Motil Cytoskeleton.

[39] Dai ZW, Zou XH, Chen GQ (2009). Poly(3-hydroxybutyrate-co-3-hydroxyhexanoate) as an injectable implant system for prevention of post-surgical tissue adhesion. Biomaterials.

[40] Ouderkirk JL, Krendel M (2014). Myosin 1e is a component of the invadosome core that contributes to regulation of invadosome dynamics. Exp Cell Res.

[41] Cui WJ, Liu Y, Zhou XL, Wang FZ, Zhang XD, Ye LH (2010). Myosin light chain kinase is responsible for high proliferative ability of breast cancer cells via anti-apoptosis involving p38 pathway. Acta Pharmacol Sin.

[42] Zhou X, Liu Y, You J, Zhang H, Zhang X, Ye L (2008). Myosin light-chain kinase contributes to the proliferation and migration of breast cancer cells through cross-talk with activated ERK1/2. Cancer Lett.

[43] Dunn TA, Chen S, Faith DA, Hicks JL, Platz EA, Chen Y, Ewing CM, Sauvageot J, Isaacs WB, De Marzo AM, Luo J (2006). A novel role of myosin VI in human prostate cancer. Am J Pathol.

[44] Küssel-Andermann P, El-Amraoui A, Safieddine S, Nouaille S, Perfettini I, Lecuit M, Cossart P, Wolfrum U, Petit C (2000). Vezatin, a novel transmembrane protein, bridges myosin VIIA to the cadherin-catenins complex. EMBO J.

[45] Omelchenko T, Hall A (2012). Myosin-IXA regulates collective epithelial cell migration by targeting RhoGAP activity to cell-cell junctions. Curr Biol.

[46] Arjonen A, Kaukonen R, Mattila E, Rouhi P, Högnäs G, Sihto H, Miller BW, Morton JP, Bucher E, Taimen P, Virtakoivu R, Cao Y, Sansom OJ (2014). Mutant p53-associated myosin-X upregulation promotes breast cancer invasion and metastasis. J Clin Invest.

[47] Zhong J, Chen Y, Wang LJ (2016). Emerging molecular basis of hematogenous metastasis in gastric cancer. World J Gastroenterol.

[48] Hart IR, Saini A (1992). Biology of tumour metastasis. Lancet.

[49] Dugina V, Khromova N, Rybko V, Blizniukov O, Shagieva G, Chaponnier C, Kopnin B, Kopnin P (2015). Tumor promotion by γ and suppression by β non-muscle actin isoforms. Oncotarget.

[50] Dugina V, Zwaenepoel I, Gabbiani G, Clément S, Chaponnier C (2009). Beta and gamma-cytoplasmic actins display distinct distribution and functional diversity. J Cell Sci.

[51] Shum MS, Pasquier E, Po’uha ST, O’Neill GM, Chaponnier C, Gunning PW, Kavallaris M (2011). γ-Actin regulates cell migration and modulates the ROCK signaling pathway. FASEB J.

[52] Tondeleir D, Lambrechts A, Müller M, Jonckheere V, Doll T, Vandamme D, Bakkali K, Waterschoot D, Lemaistre M, Debeir O, Decaestecker C, Hinz B, Staes A (2012). Cells lacking β-actin are genetically reprogrammed and maintain conditional migratory capacity. Mol Cell Proteomics.

[53] Li YR, Yang WX (2016). Myosin superfamily: the multi-functional and irreplaceable factors in spermatogenesis and testicular tumors. Gene.

[54] Mermall V, Post PL, Mooseker MS (1998). Unconventional myosins in cell movement, membrane traffic, and signal transduction. Science.

[55] Kaneko K, Satoh K, Masamune A, Satoh A, Shimosegawa T (2002). Myosin light chain kinase inhibitors can block invasion and adhesion of human pancreatic cancer cell lines. Pancreas.

[56] Jacobs K, Van Gele M, Forsyth R, Brochez L, Vanhoecke B, De Wever O, Bracke M (2010). P-cadherin counteracts myosin II-B function: implications in melanoma progression. Mol Cancer.

[57] Bai J, Uehara Y, Montell DJ (2000). Regulation of invasive cell behavior by taiman, a Drosophila protein related to AIB1, a steroid receptor coactivator amplified in breast cancer. Cell.

[58] Lo CM, Buxton DB, Chua GC, Dembo M, Adelstein RS, Wang YL (2004). Nonmuscle myosin IIb is involved in the guidance of fibroblast migration. Mol Biol Cell.

[59] Luxenburg C, Pasolli HA, Williams SE, Fuchs E (2011). Developmental roles for Srf, cortical cytoskeleton and cell shape in epidermal spindle orientation. Nat Cell Biol.

[60] Lu H, Zhao Q, Jiang H, Zhu T, Xia P, Seffens W, Aikhionbare F, Wang D, Dou Z, Yao X (2014). Characterization of ring-like F-actin structure as a mechanical partner for spindle positioning in mitosis. PLoS One.

[61] Wordeman L, Decarreau J (2016). Revisiting Actin's role in early centrosome separation. Cell Cycle.

[62] Morin X, Bellaïche Y (2011). Mitotic spindle orientation in asymmetric and symmetric cell divisions during animal development. Dev Cell.

[63] Whitehead CM, Winkfein RJ, Rattner JB (1996). The relationship of HsEg5 and the actin cytoskeleton to centrosome separation. Cell Motil Cytoskeleton.

[64] Wu X, Kocher B, Wei Q, Hammer JA (1998). Myosin Va associates with microtubule-rich domains in both interphase and dividing cells. Cell Motil Cytoskeleton.

[65] Sanger JM, Mittal B, Dome JS, Sanger JW (1989). Analysis of cell division using fluorescently labeled actin and myosin in living PtK2 cells. Cell Motil Cytoskeleton.

[66] Wang YL, Taylor DL (1979). Distribution of fluorescently labeled actin in living sea urchin eggs during early development. J Cell Biol.

[67] Cramer LP, Mitchison TJ (1995). Myosin is involved in postmitotic cell spreading. J Cell Biol.

[68] Silverman-Gavrila RV, Forer A (2001). Effects of anti-myosin drugs on anaphase chromosome movement and cytokinesis in crane-fly primary spermatocytes. Cell Motil Cytoskeleton.

[69] D’Avino PP, Giansanti MG, Petronczki M (2015). Cytokinesis in animal cells. Cold Spring Harb Perspect Biol.

[70] Mullins JM, McIntosh JR (1982). Isolation and initial characterization of the mammalian midbody. J Cell Biol.

[71] Mierzwa B, Gerlich DW (2014). Cytokinetic abscission: molecular mechanisms and temporal control. Dev Cell.

[72] Kuo TC, Chen CT, Baron D, Onder TT, Loewer S, Almeida S, Weismann CM, Xu P, Houghton JM, Gao FB, Daley GQ, Doxsey S (2011). Midbody accumulation through evasion of autophagy contributes to cellular reprogramming and tumorigenicity. Nat Cell Biol.

[73] Huang JD, Brady ST, Richards BW, Stenolen D, Resau JH, Copeland NG, Jenkins NA (1999). Direct interaction of microtubule- and actin-based transport motors. Nature.

[74] Waterman-Storer C, Duey DY, Weber KL, Keech J, Cheney RE, Salmon ED, Bement WM (2000). Microtubules remodel actomyosin networks in Xenopus egg extracts via two mechanisms of F-actin transport. J Cell Biol.

[75] Cao TT, Chang W, Masters SE, Mooseker MS (2004). Myosin-Va binds to and mechanochemically couples microtubules to actin filaments. Mol Biol Cell.

[76] Rodriguez OC, Cheney RE (2002). Human myosin-Vc is a novel class V myosin expressed in epithelial cells. J Cell Sci.

[77] Dong W, Chen X, Chen P, Yue D, Zhu L, Fan Q (2012). Inactivation of MYO5B promotes invasion and motility in gastric cancer cells. Dig Dis Sci.

[78] Müller T, Hess MW, Schiefermeier N, Pfaller K, Ebner HL, Heinz-Erian P, Ponstingl H, Partsch J, Röllinghoff B, Köhler H, Berger T, Lenhartz H, Schlenck B (2008). MYO5B mutations cause microvillus inclusion disease and disrupt epithelial cell polarity. Nat Genet.

[79] Bilder D (2004). Epithelial polarity and proliferation control: links from the Drosophila neoplastic tumor suppressors. Genes Dev.

[80] Dong W, Wang L, Shen R (2013). MYO5B is epigenetically silenced and associated with MET signaling in human gastric cancer. Dig Dis Sci.

