# Supplementary material for: Myosin Va plays essential roles in maintaining normal mitosis, enhancing tumor cell motility and viability
Source: Oncotarget. 2017 May 17;8(33):54654–71. doi: 10.18632/oncotarget.17920 (PMC5589611; doi:10.18632/oncotarget.17920)
Supplement: Supplementary file 1 [file oncotarget-08-54654-s001.pdf]

Myosin Va plays essential roles in maintaining normal mitosis, enhancing tumor cell motility and viability

SUPPLEMENTARY MATERIALS

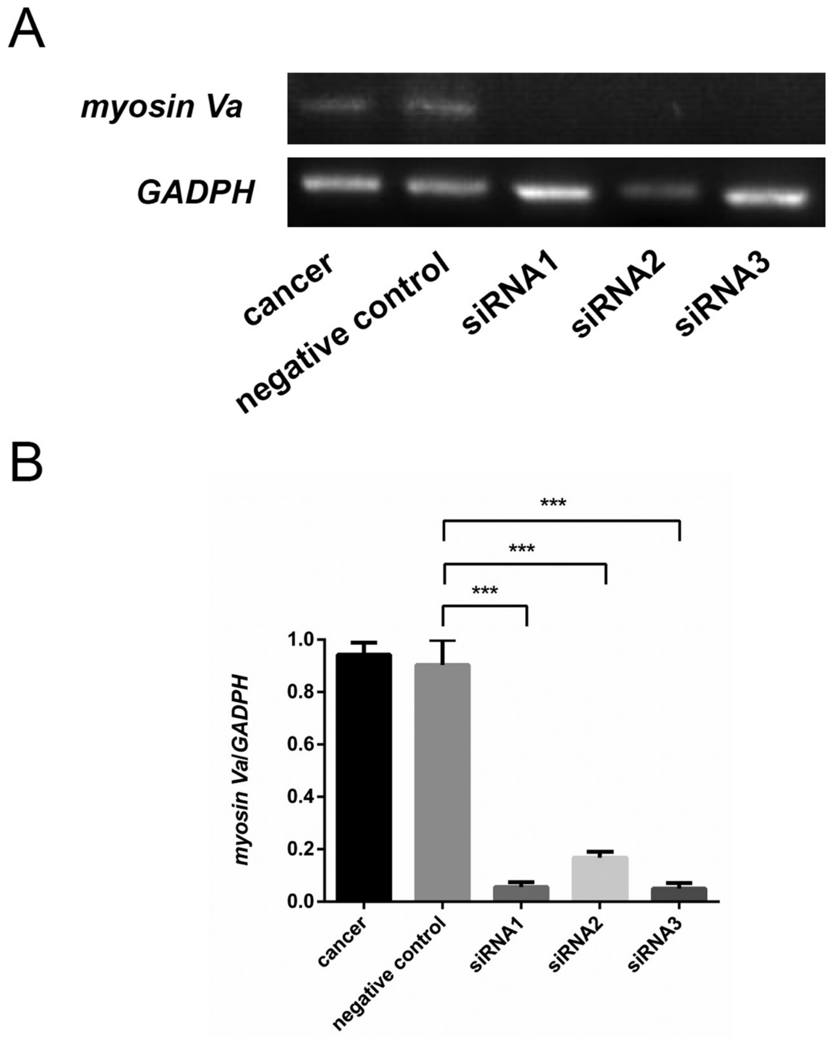

Supplementary Figure 1: In our knock-down assay, three siRNA treated groups are analyzed, and a cancer cell group serves as a contrasting group. (**A**) RT-PCR results show that siRNA knock-down has obvious effects. (**B**) Cancer and negative control groups show higher expression levels of myosin Va than siRNA treated cell groups.
